# Supplementary material for: Skew in ovarian activation depends on domicile size in phyllode-glueing thrips
Source: Sci Rep. 2018 Feb 26;8:3597. doi: 10.1038/s41598-018-21635-z (PMC5832150; doi:10.1038/s41598-018-21635-z)
Supplement: Supplementary file 1 — All datasets with field descriptions [file 41598_2018_21635_MOESM1_ESM.pdf]

Skew in ovarian activation depends on domicile size in phyllode-glueing thrips

J. D. J. Gilbert, A. Wells and S. J. Simpson

Supplementary file: all datasets used in this study

Dataset for analysis of per capita productivity versus nest volume

vol	Nest volume (mm3)
percapita	Per capita offspring
foundress	Number of foundresses
fcut	Multiple foundresses (1=no, 2=yes)

	SITE	TREE	vol	percapita	foundress	fcut
1	F	F6	606	3.7826087	11	2
2	F	F6	371.8	9.30769231	6	2
3	F	F6	232.2	7.13333333	7	2
4	F	F6	84.252	5.66666667	1	1
5	F	F6	665.184	5.54545455	5	2
6	F	F6	723.486	8.14285714	10	2
7	F	F6	222.74	4.27272727	5	2
8	F	F6	289.536	8.69230769	6	2
9	F	F6	20.352	4.14285714	3	2
10	F	F6	561.249	8.05882353	8	2
11	F	F5	243.542	7.85714286	3	2
12	F	F4	1010.1	3.27906977	21	2
13	F	F5	121.44	3	2	2
14	F	F4	180.96	5	3	2
15	F	F5	194.25	8.71428571	3	2
16	F	F5	202.244	8.71428571	3	2
17	F	F4	1236.437	4.79310345	14	2
18	F	F2	255.375	7.44444444	4	2
19	F	F4	52.38	1.66666667	1	1
20	F	F2	111.078	7.8	2	2
21	F	F4	57.834	4.14285714	3	2
22	F	F2	299.592	6.09090909	5	2
23	F	F2	441.6	9.85714286	3	2
24	F	F2	107.64	4.33333333	1	1
25	NH	NH1	185.52375	5.22222222	4	2
26	NH	NH1	442.4475	8.14285714	3	2
27	NH	NH1	147.56	13	1	1
28	NH	NH1	142.443	9.4	2	2
29	NH	NH1	62.08125	3	2	2
30	NH	NH1	89.908	5	2	2
31	NH	NH1	63.36	5.8	2	2
32	NH	NH1	396.929875	6.45454546	5	2
33	NH	NH1	773.0325	12.6363636	5	2
34	NH	NH1	425.25	11.4	2	2
35	LV	LV2	109.776875	13.8	2	2
36	LV	LV1	83.16	2.33333333	1	1
37	LV	LV1	132.066	5.66666667	1	1
38	LV	LV1	115.4925	4.33333333	1	1
39	LV	LV1	114	7	1	1

40	LV	LV1	84.64	8.33333333	1	1
41	LV	LV1	172.84	7.4	2	2
42	LV	LV2	174.741	5	2	2
43	LV	LV2	117.936	4.2	2	2
44	LV	LV2	282.295	14.3333333	1	1
45	LV	LV2	180.93	1	3	2
46	LV	LV1	44.064	12.3333333	1	1
47	LV	LV1	91.325	3.66666667	1	1
48	LV	LV1	362.232	2.77777778	4	2
49	LV	LV1	47.6625	1	1	1
50	LV	LV1	24.268125	3	1	1
51	LV	LV2	44.07425	4.33333333	1	1
52	LV	LV2	309.375	17.6666667	1	1
53	LV	LV2	573.87	4.33333333	1	1
54	LV	LV2	31.863	7	1	1
55	NH	NH1	123.75	1.28571429	3	2
56	NH	NH1	373.626	7	1	1
57	LV	LV2	84	8.33333333	1	1
58	LV	LV1	219.912	2.6	2	2
59	LV	LV1	62.8575	11.6666667	1	1
60	LV	LV2	121.8735	5	1	1
61	LV	LV2	151.632	7.57142857	3	2
62	F	F3	542.4105	10.3333333	4	2
63	F	F3	350.574	6.23809524	10	2
64	F	F3	1025.64	6.15789474	9	2
65	F	F1	211.584	2.71428571	3	2
66	F	F6	478.3725	11.1538462	6	2
67	F	F6	312.375	14.1428571	3	2
68	F	F6	117.4775	4.33333333	4	2
69	F	F6	24.99	2.33333333	1	1
70	F	F6	94.608	1.44444444	4	2
71	F	F6	90.909	0.6	2	2
72	F	F6	318.33	1	2	2
73	F	F6	64.53	2.33333333	4	2
74	F	F6	123.8705	16.1428571	3	2
75	F	F6	143.1045	1.88888889	4	2
76	F	F6	228.3525	8.33333333	4	2
77	F	F6	66.963	3.4	2	2
78	F	F6	979.0445	4.42857143	17	2
79	F	F6	908.4075	15.4	2	2
80	F	F6	259.6125	5.18181818	5	2
81	F	F6	72.712	2.81818182	5	2
82	F	F6	137.25	1.4	2	2
83	F	F6	179.55	11.8	2	2
84	F	F6	277.242	1.7826087	11	2
85	F	F6	538.98075	6.33333333	1	1

86	F	F6		241.428	2.6	2	2
87	F	F2		1039.896	3.44444444	4	2
88	F	F2		85.26	1.28571429	3	2
89	F	F2		219.7845	1	1	1
90	F	F6		137.775	6.2	2	2
91	BH		4	320.068	1.72727273	5	2
92	BH		4	436.59	0.71428571	3	2
93	BH		4	57.722	3.28571429	3	2
94	BH		3	14.094	11	1	1
95	BH		7	106.704	3.8	2	2
96	BH		4	192.276	15	1	1
97	BH		4	108.672	3	2	2
98	BH		4	212.16	9.36363636	5	2
99	BH		4	277.641	1.57142857	3	2
100	BH		4	109.56875	3	3	2
101	BH		4	66.69	3	1	1
102	BH		4	82.215	5.8	2	2
103	BH		4	112.158	4.6	2	2
104	BH		4	63.168	1	2	2
105	BH		4	100.902	0.6	2	2
106	BH		4	87.516	10.3333333	1	1
107	S		1	28.638	2.33333333	1	1
108	S		1	30.69	5	1	1
109	S		1	64.8	3.4	2	2
110	S		2	182.115	1.57142857	3	2
111	S		3	50.688	6.33333333	1	1
112	S		4	176.98625	2.2	2	2
113	S		4	105.84	1.90909091	5	2
114	S		6	63.51	2.33333333	1	1
115	S		6	109.72325	0	1	1
116	S		7	57.81	3	1	1
117	S		7	29.988	2.33333333	1	1
118	S		4	157.9725	3.66666667	4	2
119	S		4	31.05	2.6	2	2
120	S		8	33.21	7	1	1
121	S		6	98.5635	5.8	2	2
122	S		4	40.92	1.28571429	3	2
123	S		6	50.1795	4.33333333	1	1
124	NC		2	122.525	9.66666667	1	1
125	NC		2	16.8875	1	1	1
126	NC		2	40.128	3.66666667	1	1
127	NC		9	66.8	1.28571429	3	2
128	NC		13	122.4	3.66666667	1	1
129	NC		13	60	4.6	2	2
130	NC		9	169.344	7.4	2	2
131	NC		9	123.48	1.66666667	1	1

132	NC	2	46.98	5	1	1
133	NC	3	107.97	1.66666667	1	1
134	NC	3	81.4275	1.66666667	1	1
135	NC	3	104.7	3	2	2
136	NC	3	137.085	1	1	1
137	NC	3	149.04	4.2	2	2
138	NC	3	124.674375	0	1	1
139	NC	3	165.168	5	2	2
140	NC	4	79.288	4.09090909	5	2
141	NC	4	26.5	1.66666667	1	1
142	NC	11	144.626625	4.33333333	1	1
143	NC	11	214.225	3	1	1
144	F	43	39.3235	1	1	1
145	F	45	47.68	5.66666667	1	1
146	F	27	77.29	12.3333333	1	1
147	F	35	45.144	4.33333333	1	1
148	F	26	98.3825	3	2	2
149	F	10	55.176	9	1	1
150	F	29	53.7425	3.66666667	1	1
151	F	7	136.249	7.8	2	2
152	F	11	95.03	5	2	2
153	F	34	47.41125	8.33333333	1	1
154	F	45	51.0245	0	2	2
155	F	45	222.75	1.28571429	3	2
156	F		49.8465	0	1	1
157	F		46.189	2.33333333	1	1
158	F		79.38	2.77777778	4	2
159	F	45	148.708	2.63636364	5	2
160	F	43	78.3475	5	1	1
161	F	37	47.799	6.33333333	1	1
162	F	45	53.34	4.33333333	1	1
163	F	26	29.79675	3	1	1
164	F	40	109.8125	0.6	7	2
165	F	37	114.71825	7	1	1
166	F	20	96.432	0	1	1
167	F	20	62.244	3	1	1
168	F	34	41.664	3.66666667	1	1
169	F	47	106.191	0.6	2	2
170	F	9	56.43	5.66666667	1	1
171	F	48	158.055	1.4	2	2
172	F	37	89.28	1.66666667	1	1
173	F	6	129.36	9.66666667	1	1
174	F	2	136.08	0.55555556	4	2
175	F	38	69.35	4.33333333	1	1
176	F	10	47.4875	5.66666667	1	1
177	F	29	24.31	0	2	2

178	F	9	42.75	1.57142857	3	2
179	F	26	57.2565	4.33333333	1	1
180	F	33	49.761	3	1	1
181	F	26	80.85	1.66666667	1	1
182	F	45	45.6665	1.66666667	1	1
183	F	43	29.375	1.66666667	1	1
184	F	9	37.43	3.66666667	1	1
185	F	45	119.85	1	1	1
186	F	34	46.8	3.66666667	1	1
187	F	46	44.655	1	1	1
188	F	28	59.1465	1	1	1
189	F	22	204.867	6.33333333	1	1
190	FX	6	280.899	0.71428571	3	2
191	FX	6	280.899	0.6	2	2
192	FX	17	38.34	3	1	1
193	F	26	134.82	5	1	1
194	FX	19	220.48	6.33333333	1	1
195	FX	7	190.736	3.44444444	4	2
196	FX	1	364.1	1.66666667	1	1
197	FX	12	303.282	6.2	2	2
198	FX	12	348.936	7.44444444	4	2
199	FX	12	43.092	7	1	1
200	FX	12	383.04	5.28571429	3	2
201	FX	18	262.752	9	3	2
202	FX	18	32.89	8.33333333	1	1
203	FX	17	244.218	4.6	2	2
204	FX	9	209.952	6.6	2	2
205	FX	12	190.35	5	2	2
206	FX	14	258.552	2.33333333	4	2
207	FX	17	203.456	2.33333333	1	1
208	FX	6	240.352	5	1	1
209	FX	17	256.23	8.2	2	2
210	FX	14	867.3	10.0769231	6	2
211	FX	14	47.824	3.8	2	2
212	FX	13	137.214	2.6	2	2
213	FX	22	102.114	3.66666667	1	1
214	FX	7	190.736	1.54545455	5	2
215	FX	12	121.716	7	1	1
216	FX	7	47.88	5	1	1
217	FX	25	51.744	11.6666667	1	1
218	FX	4	139.392	3.66666667	1	1
219	FX	6	160.65	6.33333333	1	1
220	FX	9	66.24	5.66666667	1	1
221	FX	9	66.447	5	1	1
222	F	8	157.248	8.33333333	1	1
223	F	26	73.6	3.66666667	1	1

224	FX		9	60.45	3.4	2	2
225	FX		4	117.35525	9	4	2
226	FX		4	134.524	3.28571429	3	2
227	F			15.68	0	1	1
228	F		3	105.84	0	2	2
229	F		3	50.544	5.66666667	1	1
230	F		3	106.56	8.6	2	2
231	F		4	220.704	8.27272727	5	2
232	F		4	65.52	13.6666667	1	1
233	F		3	602.7	5.18181818	5	2
234	F		3	881.328	7.90909091	5	2
235	F		3	64.438	6.2	2	2
236	F		3	558.45	6.81818182	5	2
237	F	2X		45.318	3	1	1
238	F	2X		153.171	4.33333333	1	1
239	F	2X		67.914	3	1	1
240	F	2X		181.764	1.8	2	2
241	F	1X		337.824	15	4	2
242	F	1X		174.42	7	1	1
243	F	1X		203.796	1	1	1
244	F	5X		416.936	0	1	1
245	F	5X		543.348	0	1	1
246	F	5X		146.16	3.66666667	4	2
247	F	5X		79.632	11	1	1
248	F	5X		68.418	7	1	1
249	F	5X		135.936	10.6	2	2
250	F		1	894.608	0.77777778	4	2
251	F		1	583.792	0.33333333	4	2
252	F		1	1178.931	0.2631579	9	2
253	F		1	68.068	5	1	1
254	F		1	70.992	0	1	1
255	F		1	98.868	0	1	1
256	F		1	580.5	0	2	2
257	F		3	569.052	0	2	2
258	F		3	181.598	1.66666667	1	1
259	F		3	24.9	0	1	1
260	F		1	173.34	0	2	2
261	F		3	280.761	11.1333333	7	2
262	F		1	99.792	4.33333333	1	1
263	F		3	155.788	9.66666667	1	1
264	F		1	175.77	4.2	2	2
265	F		3	47.025	6.6	2	2
266	F		1	439.296	9.28571429	3	2
267	F		4	828.8	2.6969697	16	2
268	F		4	478.007	0.47826087	11	2
269	F		4	491.92	0.15789474	9	2

270	F	4	51.48	0	1	1
271	F	4	265.734	2.2	7	2
272	F	4	365.904	2.52380952	10	2
273	F	4	211.344	2.71428571	3	2
274	F	6	105.7	3	4	2
275	F	6	149.85	19.6666667	1	1
276	F	6	51.48	0	2	2
277	F	6	565.248	6.2	2	2
278	F	6	557.235	15.6666667	1	1
279	F	3	359.64	0.6	2	2
280	F	3	287.826	0	3	2
281	F	3	227.64	2.42857143	3	2
282	F	3	228.69	2.14285714	3	2
283	F	4	866.151	5	7	2
284	F	4	992.64	9.36363636	5	2
285	F	4	1322.615	0.89189189	18	2
286	F	4	473.34	5	5	2
287	F	4	168.609	1.28571429	3	2
288	F	4	1408.035	2.61904762	10	2
289	F	4	454.656	0.65714286	17	2
290	F	3	602.25	0	3	2
291	F	3	722.228	2.38461539	6	2
292	F	3	659.532	0	1	1
293	F	3	118.218	1.66666667	1	1
294	F	6	381.216	8.33333333	1	1
295	F	6	104.64	7.30769231	6	2
296	F	6	169.46	14.7142857	3	2
297	F	6	148.2	5.4	2	2
298	F	3	492.1	4.2	2	2
299	F	3	251.1	0	1	1
300			114.675	11	1	1
301			104.625	0	1	1
302			107.632	9	1	1
303	F	3	558.9	0	4	2
304	F	3	445.725	0.77777778	4	2
305	F	3	336.168	3.15384615	6	2
306	F	6	304.64	2.84615385	6	2
307	F	6	256.5	0	1	1
308	F	6	722.43	7.26666667	7	2
309	F	6	107.38	1	1	1
310	F	3	141.36	1	1	1
311	F	2	454.86	5.44444444	4	2
312	F	2	109.836	0	1	1
313	F	2	209.88	11.7692308	6	2
314	F	2	919.82	7.60869565	11	2
315	F	6	174.24	0	2	2

Dataset for individual level analysis of oocyte volume in reproductives versus nest volume

nestvol	Nest volume, mm3
females	Total number of foundresses in the domicile
nrbin	Nonreproductives present in the nest
pronotum	Pronotum width in um
oovol	Volume of developing oocytes in dissected ovary, um3

	ID.NEST	nestvol	females	pronotum	nrbin	oovol
1	I2-7	73.44	2	320	FALSE	27.0993782
2	I1-2	83.232	1	320	FALSE	7.06858347
3	F16.0.2	281.799	4	340	TRUE	0.90477868
4	F16.0.2	281.799	4	320	TRUE	7.81942412
5	F16.0.2	281.799	4	360	TRUE	1.39957953
6	FX.1	134.64	2	320	TRUE	0.60318579
7	F48.1	106.64325	3	280	TRUE	7.16911444
8	F28.2	73.8675	1	300	FALSE	16.512211
9	F48.2	75.992	2	260	TRUE	1.10584061
10	I1-12	55.176	1	320	FALSE	2.49206837
11	I1-21	53.7425	1	340	FALSE	12.8821007
12	I2-1	136.249	2	320	FALSE	5.46008803
13	I2-1	136.249	2	320	FALSE	11.5045123
14	I1-14	95.03	2	290	TRUE	1.88495559
15	I3-1	222.75	3	300	FALSE	4.73752172
16	I3-1	222.75	3	310	FALSE	1.00530965
17	I3-1	222.75	3	280	FALSE	3.43061918
18	I2-9	79.38	4	300	TRUE	1.41371669
19	I2-9	79.38	4	300	TRUE	3.85159259
20	I2-9	79.38	4	320	TRUE	1.57079633
21	I4-1	148.708	5	340	TRUE	2.26596795
22	I1-43	78.3475	1	300	FALSE	8.25610549
23	I1-64	16.7025	1	320	FALSE	1.25663706
24	I1-34	47.799	1	310	FALSE	1.41371669
25	I1-58	53.34	1	290	FALSE	1.88495559
26	I1-37	109.8125	7	310	TRUE	0.80424772
27	I1-37	109.8125	7	300	TRUE	1.44513262
28	I1-33	114.71825	1	290	FALSE	0.39584067
29	I1-9	62.244	1	320	FALSE	0.60318579
30	I1-28	41.664	1	300	FALSE	1.85982285
31	I1-63	106.191	2	320	FALSE	1.25663706
32	I1-63	106.191	2	300	FALSE	1.09955743
33	I1-3	56.43	1	320	FALSE	1.90066356
34	I2-14	158.055	2	290	FALSE	1.41371669
35	I2-14	158.055	2	320	FALSE	1.57079633
36	I1-68	89.28	1	300	FALSE	10.9327424
37	F6.0.1	129.36	1	280	FALSE	0.60318579
38	F2.0.1	136.08	4	360	TRUE	2.26194671

39	F38.0.1	69.35	1	320	FALSE	2.17712371
40	F9.0.1	42.75	3	340	TRUE	1.20637158
41	I1-48	57.2565	1	300	FALSE	0.60318579
42	I1-57	45.6665	1	310	FALSE	2.17398212
43	I1-50	119.85	1	310	FALSE	1.64933614
44	I1-70	53.58	1	320	FALSE	1.14511052
45	FX9-20	415.436	3	320	FALSE	4.64563014
46	FX9-20	415.436	3	300	FALSE	3.5060174
47	FX9-20	415.436	3	320	FALSE	3.61911474
48	FX6-7	240.352	2	340	TRUE	15.2893461
49	FX9-10	139.36	2	340	TRUE	8.90798597
50	FX17-12	203.456	2	300	FALSE	1.09955743
51	FX17-12	203.456	2	300	FALSE	18.1364144
52	FX13-1	150.304	2	280	TRUE	9.93842836
53	FX1-1	380.16	2	290	FALSE	6.38371627
54	FX1-1	380.16	2	300	FALSE	24.7431837
55	FX11-2	248.009	4	300	FALSE	14.5000209
56	FX11-2	248.009	4	280	FALSE	3.29867229
57	FX11-2	248.009	4	360	FALSE	5.33756592
58	FX11-2	248.009	4	320	FALSE	17.7060162
59	F8-3	24.15	2	330	FALSE	5.81194641
60	FX9-15	277.35	4	300	TRUE	1.25663706
61	FX9-15	277.35	4	320	TRUE	2.43473431
62	FX9-15	277.35	4	340	TRUE	1.01787602
63	FX6-5A	280.899	2	280	FALSE	10.3955301
64	FX6-5A	280.899	2	260	FALSE	13.9133285
65	FX6-5B	280.899	2	340	FALSE	16.5467685
66	FX6-5B	280.899	2	320	FALSE	2.14727858
67	FX12-6	582.153	6	300	TRUE	6.00672515
68	FX12-6	582.153	6	320	TRUE	6.98376047
69	FX12-6	582.153	6	320	TRUE	6.44026494
70	FX12-6	582.153	6	300	TRUE	6.20464549
71	FX12-6	582.153	6	300	TRUE	2.10486708
72	FX12-1	150.304	5	290	FALSE	10.951592
73	FX12-1	150.304	5	300	FALSE	13.2543794
74	FX12-1	150.304	5	290	FALSE	15.0136713
75	FX12-1	150.304	5	280	FALSE	3.00336258
76	FX12-1	150.304	5	260	FALSE	5.52134909
77	FX12-5	61.75	3	280	FALSE	20.0622107
78	FX12-5	61.75	3	310	FALSE	11.8123884
79	FX12-5	61.75	3	330	FALSE	10.9610168
80	FX17-14	38.34	1	300	FALSE	10.4897779
81	FX19-4	220.48	1	290	FALSE	11.9796782
82	FX7-4	190.736	4	340	FALSE	2.88084046
83	FX7-4	190.736	4	300	FALSE	5.52920307
84	FX7-4	190.736	4	300	FALSE	11.6207512

85	FX7-4	190.736	4	240	FALSE	4.41707927
86	FX1-2	364.1	1	320	FALSE	13.2952201
87	FX9-17	268.92	5	260	FALSE	13.5716803
88	FX9-17	268.92	5	280	FALSE	19.4778745
89	FX9-17	268.92	5	300	FALSE	16.7038481
90	FX9-17	268.92	5	260	FALSE	18.2652197
91	FX9-18	403	3	320	FALSE	10.0530965
92	FX9-18	403	3	300	FALSE	8.65508776
93	FX9-18	403	3	280	FALSE	0.92362824
94	FX9-14	145.86	2	320	FALSE	9.61327352
95	FX12-18	303.282	2	300	FALSE	16.7988813
96	FX12-18	303.282	2	280	FALSE	8.44303026
97	FX12-13	348.936	4	350	FALSE	30.0524753
98	FX12-13	348.936	4	310	FALSE	7.11256577
99	FX12-13	348.936	4	300	FALSE	6.90522065
100	FX12-13	348.936	4	310	FALSE	17.7876976
101	FX12-14	383.04	3	300	FALSE	10.6829858
102	FX12-14	383.04	3	310	FALSE	5.36584025
103	FX12-14	383.04	3	340	FALSE	28.481679
104	FX12-14B	43.092	1	320	FALSE	7.70082899
105	FX18-1	405.108	3	290	FALSE	19.3899099
106	FX18-1	405.108	3	300	FALSE	4.91345091
107	FX18-1	405.108	3	300	FALSE	9.79784209
108	FX18-1B	32.89	1	310	FALSE	10.0782292
109	FX9-13	295.659	2	330	FALSE	12.2168684
110	FX9-13	295.659	2	350	FALSE	18.1214918
111	FX4-9	934.912	5	320	FALSE	20.5774319
112	FX4-9	934.912	5	320	FALSE	11.2390477
113	FX17-15	244.218	2	300	FALSE	1.00530965
114	FX17-15	244.218	2	300	FALSE	1.7090264
115	FX4-9	934.912	5	280	FALSE	8.96924703
116	FX4-9	934.912	5	310	FALSE	6.05699064
117	FX4-9	934.912	5	340	FALSE	11.1338044
118	FX13-5	212.8	2	300	TRUE	0.95504417
119	FX13-9	347.776	5	310	FALSE	3.1667254
120	FX13-9	347.776	5	300	FALSE	3.39292007
121	FX13-9	347.776	5	290	FALSE	4.51289785
122	FX13-9	347.776	5	320	FALSE	2.94053072
123	FX13-9	347.776	5	300	FALSE	1.57079633
124	FX9-1	209.952	2	300	FALSE	12.2773441
125	FX9-1	209.952	2	330	FALSE	16.5781844
126	FX12-3	190.35	2	330	FALSE	16.3614145
127	FX12-3	190.35	2	320	FALSE	16.1619234
128	FX14-3	258.552	4	260	TRUE	1.17809725
129	FX17-13	256.23	2	290	FALSE	0.53721234
130	FX17-13	256.23	2	320	FALSE	1.41371669

131	FX14-1	867.3	8	280	TRUE	0.60318579
132	FX14-1	867.3	8	300	TRUE	1.57079633
133	FX14-1	867.3	8	320	TRUE	0.80424772
134	FX14-1	867.3	8	280	TRUE	1.96349541
135	FX14-1	867.3	8	280	TRUE	1.01787602
136	FX14-1	867.3	8	280	TRUE	1.88495559
137	FX14-1	867.3	8	300	TRUE	1.82212374
138	FX14-1A	47.824	1	320	FALSE	8.49800813
139	F8-3	24.15	2	310	FALSE	4.24115008
140	FX12-7	203.456	1	320	FALSE	19.4605957
141	FX23-1	102.114	1	290	FALSE	12.1736715
142	FX7-1	190.736	5	310	FALSE	17.4861047
143	FX7-1	190.736	5	340	FALSE	11.4126207
144	FX7-1	190.736	5	300	FALSE	15.7173881
145	FX7-1	190.736	5	320	FALSE	8.19955683
146	FX7-1	190.736	5	310	FALSE	11.784114
147	FX9-17	268.92	5	250	FALSE	5.18834027

excluded_for_analysis
FALSE
FALSE
FALSE
FALSE
FALSE
FALSE
FALSE
FALSE
FALSE
FALSE
FALSE
FALSE
FALSE
FALSE
FALSE
FALSE
FALSE
FALSE
FALSE
FALSE
FALSE
FALSE
FALSE
FALSE
FALSE
TRUE
TRUE
FALSE
FALSE
FALSE
FALSE
FALSE
FALSE
FALSE
FALSE
FALSE
FALSE
FALSE

FALSE
FALSE
FALSE
FALSE
FALSE
FALSE
FALSE
FALSE
FALSE
FALSE
FALSE
FALSE
FALSE
FALSE
FALSE
FALSE
FALSE
FALSE
FALSE
FALSE
FALSE
FALSE
FALSE
FALSE
FALSE
FALSE
FALSE
FALSE
TRUE
TRUE
TRUE
TRUE
TRUE
FALSE
FALSE
FALSE
FALSE
FALSE
FALSE
FALSE
FALSE
FALSE
FALSE
FALSE
FALSE
FALSE

FALSE
FALSE
FALSE
FALSE
FALSE
FALSE
FALSE
FALSE
FALSE
FALSE
FALSE
FALSE
FALSE
FALSE
FALSE
FALSE
FALSE
FALSE
FALSE
FALSE
FALSE
FALSE
FALSE
FALSE
FALSE
FALSE
FALSE
FALSE
FALSE
FALSE
FALSE
FALSE
FALSE
FALSE
FALSE
FALSE
FALSE
FALSE
FALSE
FALSE
FALSE
FALSE
FALSE
FALSE
FALSE
FALSE

TRUE
TRUE
TRUE
TRUE
TRUE
TRUE
TRUE
FALSE
FALSE
FALSE
FALSE
FALSE
FALSE
FALSE
FALSE
FALSE
FALSE

Dataset for nest-level analysis of oocyte volume versus nest volume

vol	Nest volume, mm3
females	Foundresses in nest
nrbin	Nonreproductives present in nest
rep.oovol	Mean oocyte volume of reproductive females in nest

	id	vol	females	nrbin	rep.oovol	excluded_for
1	F10-1	NA	1	TRUE	NA	NA
2	F10.0.1	47.4875	1	TRUE	NA	FALSE
3	F11-2	NA	0	NA	NA	NA
4	F11.2	NA	2	FALSE	20.445485	NA
5	F14.1	NA	0	NA	NA	NA
6	F16.0.2	281.799	4	TRUE	3.37459411	FALSE
7	F17-8	NA	0	NA	NA	NA
8	F2.0.1	136.08	4	TRUE	2.26194671	FALSE
9	F22.0.1A	204.867	0	NA	NA	FALSE
10	F26-3	NA	1	FALSE	1.80641578	NA
11	F26-5X	134.82	0	NA	NA	FALSE
12	F28.1	NA	2	FALSE	7.46442415	NA
13	F28.2	73.8675	1	FALSE	16.512211	FALSE
14	F33.1	NA	1	FALSE	14.815751	NA
15	F34.0.1	NA	1	FALSE	34.9533599	NA
16	F36.1	NA	2	TRUE	NA	NA
17	F36.2	NA	2	FALSE	7.29791973	NA
18	F36.3	64.8	0	NA	NA	FALSE
19	F37.1	NA	1	TRUE	NA	NA
20	F38.0.1	69.35	1	FALSE	2.17712371	FALSE
21	F39.1	NA	1	TRUE	NA	NA
22	F40-1B	NA	1	TRUE	NA	NA
23	F47-3	NA	0	NA	NA	NA
24	F48.1	106.64325	3	TRUE	7.16911444	FALSE
25	F48.2	75.992	2	TRUE	1.10584061	FALSE
26	F6.0.1	129.36	1	FALSE	0.60318579	FALSE
27	F7.0.1	69.356625	0	NA	NA	FALSE
28	F7.0.2	NA	1	TRUE	NA	NA
29	F8-1	NA	1	FALSE	7.30263212	NA
30	F8-3	24.15	2	FALSE	5.02654825	FALSE
31	F9.0.1	42.75	3	TRUE	1.20637158	FALSE
32	FX.1	134.64	2	TRUE	0.60318579	FALSE
33	FX1-1	380.16	2	FALSE	15.56345	FALSE
34	FX1-2	364.1	1	FALSE	13.2952201	FALSE
35	FX11-2	248.009	4	FALSE	10.2105688	FALSE
36	FX11-3	NA	1	FALSE	6.32481141	NA
37	FX12-1	150.304	5	FALSE	9.54887087	FALSE
38	FX12-10	NA	0	NA	NA	NA
39	FX12-12	NA	0	NA	NA	NA

40	FX12-13	348.936	4	FALSE	15.4644898	FALSE
41	FX12-14	383.04	3	FALSE	14.8435017	FALSE
42	FX12-14B	43.092	1	FALSE	7.70082899	FALSE
43	FX12-16	NA	0	NA	NA	NA
44	FX12-18	303.282	2	FALSE	12.6209558	FALSE
45	FX12-2	NA	3	FALSE	6.39366465	NA
46	FX12-21	NA	1	FALSE	12.8271228	NA
47	FX12-22	NA	0	NA	NA	NA
48	FX12-3	190.35	2	FALSE	16.261669	FALSE
49	FX12-5	61.75	3	FALSE	14.2785386	FALSE
50	FX12-7	203.456	1	FALSE	19.4605957	FALSE
51	FX12-9	NA	1	FALSE	3.76676959	NA
52	FX13-1	150.304	2	TRUE	9.93842836	FALSE
53	FX13-12	NA	2	FALSE	2.43473431	NA
54	FX13-4	NA	2	FALSE	25.0742291	NA
55	FX13-4B	NA	2	FALSE	1.72787596	NA
56	FX13-5	212.8	2	TRUE	0.95504417	FALSE
57	FX13-9	347.776	5	FALSE	3.11677407	FALSE
58	FX14-1A	47.824	1	FALSE	8.49800813	FALSE
59	FX14-3	258.552	4	TRUE	1.17809725	FALSE
60	FX17-11	NA	2	FALSE	3.12431389	NA
61	FX17-12	203.456	2	FALSE	9.61798591	FALSE
62	FX17-13	256.23	2	FALSE	0.97546452	FALSE
63	FX17-14	38.34	1	FALSE	10.4897779	FALSE
64	FX17-15	244.218	2	FALSE	1.35716803	FALSE
65	FX17-3	NA	1	FALSE	14.356293	NA
66	FX18-1	405.108	3	FALSE	11.3670676	FALSE
67	FX18-1B	32.89	1	FALSE	10.0782292	FALSE
68	FX18-2	NA	1	FALSE	1.40743351	NA
69	FX18-3	NA	2	FALSE	13.9141139	NA
70	FX18-5	NA	1	FALSE	16.1100871	NA
71	FX19-4	220.48	1	FALSE	11.9796782	FALSE
72	FX22-1	NA	0	NA	NA	NA
73	FX23-1	102.114	1	FALSE	12.1736715	FALSE
74	FX24-1	NA	1	FALSE	1.60849544	NA
75	FX25-1	NA	4	FALSE	15.26009	NA
76	FX26-2	NA	3	FALSE	13.4488964	NA
77	FX26-4	NA	2	FALSE	7.79743297	NA
78	FX27-5A	NA	1	TRUE	NA	NA
79	FX4-1	NA	1	FALSE	3.92070763	NA
80	FX4-10	NA	0	NA	NA	NA
81	FX4-6	NA	4	FALSE	11.7558397	NA
82	FX4-7	NA	3	FALSE	8.28280903	NA
83	FX4-9	934.912	5	FALSE	11.5953043	TRUE
84	FX5-1	NA	2	TRUE	3.41805281	NA
85	FX5-3	NA	0	NA	NA	NA

86	FX5-4	NA	1	TRUE	NA	NA
87	FX6-1	NA	1	FALSE	12.6920343	NA
88	FX6-5A	280.899	2	FALSE	12.1544293	FALSE
89	FX6-5B	280.899	2	FALSE	9.34702354	FALSE
90	FX6-7	240.352	2	TRUE	15.2893461	FALSE
91	FX7-1	190.736	5	FALSE	12.9199569	FALSE
92	FX7-1B	NA	0	NA	NA	NA
93	FX7-4	190.736	4	FALSE	6.11196851	FALSE
94	FX7-7	97.944	3	TRUE	NA	FALSE
95	FX7-8	NA	1	FALSE	9.58499919	NA
96	FX8-3	NA	1	TRUE	NA	NA
97	FX9-1	209.952	2	FALSE	14.4277643	FALSE
98	FX9-10	139.36	2	TRUE	8.90798597	FALSE
99	FX9-11	NA	1	FALSE	16.8405074	NA
100	FX9-13	295.659	2	FALSE	15.1691801	FALSE
101	FX9-14	145.86	2	FALSE	13.3769015	FALSE
102	FX9-14B	NA	1	FALSE	26.434146	NA
103	FX9-15	277.35	4	TRUE	1.56974913	FALSE
104	FX9-16	NA	0	NA	NA	NA
105	FX9-17	268.92	5	FALSE	14.6413926	FALSE
106	FX9-18	403	3	FALSE	6.5439375	FALSE
107	FX9-20	415.436	3	FALSE	3.92358743	FALSE
108	FX9-3	NA	3	FALSE	9.25879715	NA
109	FX9-5	119.6	0	NA	NA	FALSE
110	I1-12	55.176	1	FALSE	2.49206837	FALSE
111	I1-13	NA	1	TRUE	NA	NA
112	I1-14	95.03	2	TRUE	1.88495559	FALSE
113	I1-16	37.43	0	NA	NA	FALSE
114	I1-17	96.432	1	TRUE	NA	FALSE
115	I1-18	59.1465	0	NA	NA	FALSE
116	I1-2	83.232	1	FALSE	7.06858347	FALSE
117	I1-20	12.42	0	NA	NA	FALSE
118	I1-21	53.7425	1	FALSE	12.8821007	FALSE
119	I1-24	49.761	0	NA	NA	FALSE
120	I1-27	83.475	0	NA	NA	FALSE
121	I1-28	41.664	1	FALSE	1.85982285	FALSE
122	I1-29	47.41125	1	FALSE	6.70887111	FALSE
123	I1-3	56.43	1	FALSE	1.90066356	FALSE
124	I1-33	114.71825	1	FALSE	0.39584067	FALSE
125	I1-34	47.799	1	FALSE	1.41371669	FALSE
126	I1-36	8.4245	0	NA	NA	FALSE
127	I1-39	29.375	0	NA	NA	FALSE
128	I1-41	39.494	1	TRUE	NA	FALSE
129	I1-42	87.21	0	NA	NA	FALSE
130	I1-43	78.3475	1	FALSE	8.25610549	FALSE
131	I1-45	NA	1	TRUE	NA	NA

132	I1-46	46.8	0	NA	NA	FALSE
133	I1-47	50.4	1	TRUE	NA	FALSE
134	I1-48	57.2565	1	FALSE	0.60318579	FALSE
135	I1-49	78.32175	1	TRUE	NA	FALSE
136	I1-50	119.85	1	FALSE	1.64933614	FALSE
137	I1-51	27.846	1	TRUE	NA	FALSE
138	I1-53	NA	1	TRUE	NA	NA
139	I1-54	44.655	0	NA	NA	FALSE
140	I1-56	24.31	2	TRUE	NA	FALSE
141	I1-57	45.6665	1	FALSE	2.17398212	FALSE
142	I1-58	53.34	1	FALSE	1.88495559	FALSE
143	I1-59	46.189	0	NA	NA	FALSE
144	I1-6	29.79675	1	TRUE	NA	FALSE
145	I1-60	NA	0	NA	NA	NA
146	I1-63	106.191	2	FALSE	1.17809725	FALSE
147	I1-64	16.7025	1	FALSE	1.25663706	FALSE
148	I1-68	89.28	1	FALSE	10.9327424	FALSE
149	I1-69	49.8465	0	NA	NA	FALSE
150	I1-7	80.85	0	NA	NA	FALSE
151	I1-70	53.58	1	FALSE	1.14511052	FALSE
152	I1-9	62.244	1	FALSE	0.60318579	FALSE
153	I2-1	136.249	2	FALSE	8.48230017	FALSE
154	I2-11	51.0245	2	TRUE	NA	FALSE
155	I2-14	158.055	2	FALSE	1.49225651	FALSE
156	I2-2	98.3825	1	FALSE	13.2323883	FALSE
157	I2-7	73.44	2	FALSE	19.9114142	FALSE
158	I2-9	79.38	4	TRUE	2.27870187	FALSE
159	I3-1	222.75	3	FALSE	3.05781685	FALSE
160	I4-1	148.708	5	TRUE	2.26596795	FALSE
161	I4-1A	NA	1	TRUE	NA	NA

_analysis

Dataset for individual level analysis of reproductive status versus nest volume

nestvol	Nest volume, mm3
females	Foundresses in nest
INDIVIDUAL	Individual identifier
pronotum	Pronotum width (um)
DEV.OOCYTE	Number of developing oocytes
oocyte	Volume of devloping oocytes (um3)

	ID.NEST	nestvol	females	INDIVIDUAL	pronotum	DEV.OOCYTE
1	I2-7	73.44	2	1	320	3
2	F7.0.1	69.356625	0	1	320	NA
3	I1-2	83.232	1	1	320	1
4	F36.3	64.8	0	1	340	NA
5	F16.0.2	281.799	4	1	320	NA
6	F16.0.2	281.799	4	2	300	NA
7	F16.0.2	281.799	4	3	340	1
8	F16.0.2	281.799	4	4	320	5
9	F16.0.2	281.799	4	5	360	1
10	F16.0.2	281.799	4	6	320	0
11	FX.1	134.64	2	1	330	NA
12	FX.1	134.64	2	2	320	0
13	FX.1	134.64	2	3	340	0
14	F48.1	106.64325	3	1	320	0
15	F48.1	106.64325	3	2	280	2
16	F48.1	106.64325	3	X	330	0
17	F28.2	73.8675	1	1	300	2
18	F48.2	75.992	2	1	300	0
19	F48.2	75.992	2	2	260	1
20	I1-12	55.176	1	1	320	2
21	I1-21	53.7425	1	1	340	2
22	I2-1	136.249	2	1	320	2
23	I2-1	136.249	2	2	320	3
24	I1-14	95.03	2	1	320	0
25	I1-14	95.03	2	2	290	1
26	I3-1	222.75	3	1	300	3
27	I3-1	222.75	3	2	310	1
28	I3-1	222.75	3	3	280	3
29	I1-69	49.8465	0	1	300	NA
30	I1-59	46.189	0	1	320	NA
31	I2-9	79.38	4	1	300	1
32	I2-9	79.38	4	2	300	2
33	I2-9	79.38	4	3	300	0
34	I2-9	79.38	4	4	320	1
35	I4-1	148.708	5	1	340	1
36	I4-1	148.708	5	2	260	0
37	I4-1	148.708	5	3	290	0

38	I4-1	148.708	5	4	300	0
39	I4-1	148.708	5	5	280	0
40	I1-43	78.3475	1	1	300	3
41	I1-64	16.7025	1	1	320	1
42	I1-34	47.799	1	1	310	1
43	I1-58	53.34	1	1	290	1
44	I1-6	29.79675	1	1	330	0
45	I1-37	109.8125	7	1	320	0
46	I1-37	109.8125	7	2	310	1
47	I1-37	109.8125	7	3	320	0
48	I1-37	109.8125	7	4	300	2
49	I1-37	109.8125	7	5	320	0
50	I1-37	109.8125	7	6	300	0
51	I1-37	109.8125	7	7	300	0
52	I1-36	8.4245	0	1	300	NA
53	I1-33	114.71825	1	1	290	1
54	I1-17	96.432	1	1	340	0
55	I1-9	62.244	1	1	320	1
56	I1-47	50.4	1	1	320	0
57	I1-28	41.664	1	1	300	2
58	I1-63	106.191	2	1	320	1
59	I1-63	106.191	2	2	300	1
60	I1-3	56.43	1	1	320	1
61	I2-14	158.055	2	1	290	1
62	I2-14	158.055	2	2	320	1
63	I1-68	89.28	1	1	300	3
64	I1-41	39.494	1	1	310	0
65	F6.0.1	129.36	1	1	280	1
66	F2.0.1	136.08	4	1	340	0
67	F2.0.1	136.08	4	2	360	1
68	F2.0.1	136.08	4	3	330	0
69	F2.0.1	136.08	4	4	330	0
70	F38.0.1	69.35	1	1	320	2
71	F10.0.1	47.4875	1	1	360	0
72	I1-56	24.31	2	1	320	0
73	I1-56	24.31	2	2	340	0
74	F9.0.1	42.75	3	1	340	2
75	F9.0.1	42.75	3	2	300	0
76	F9.0.1	42.75	3	3	300	0
77	I1-48	57.2565	1	1	300	1
78	I1-51	27.846	1	1	300	0
79	I1-24	49.761	0	1	360	NA
80	I1-7	80.85	0	1	300	NA
81	I1-57	45.6665	1	1	310	2
82	I1-39	29.375	0	1	290	NA
83	I1-16	37.43	0	1	300	NA

84	I1-50	119.85	1	1	310	1
85	I1-49	78.32175	1	1	270	0
86	I1-46	46.8	0	1	340	NA
87	I1-27	83.475	0	1	310	NA
88	I1-20	12.42	0	1	320	NA
89	I1-54	44.655	0	1	310	NA
90	I1-18	59.1465	0	1	300	NA
91	I1-42	87.21	0	1	300	NA
92	I1-70	53.58	1	1	320	1
93	FX9-20	415.436	3	1	320	1
94	FX9-20	415.436	3	2	300	1
95	FX9-20	415.436	3	3	320	1
96	FX6-7	240.352	2	1	340	0
97	FX6-7	240.352	2	2	340	4
98	FX9-10	139.36	2	1	350	0
99	FX9-10	139.36	2	2	340	2
100	FX17-12	203.456	2	1	300	1
101	FX17-12	203.456	2	2	300	2
102	FX13-1	150.304	2	1	280	3
103	FX13-1	150.304	2	1	300	0
104	FX1-1	380.16	2	1	290	2
105	FX1-1	380.16	2	2	300	3
106	FX11-2	248.009	4	1	300	2
107	FX11-2	248.009	4	2	280	1
108	FX11-2	248.009	4	3	360	2
109	FX11-2	248.009	4	4	320	3
110	F8-3	24.15	2	1	330	3
111	FX9-15	277.35	4	1	300	0
112	FX9-15	277.35	4	2	300	1
113	FX9-15	277.35	4	3	320	1
114	FX9-15	277.35	4	4	340	1
115	FX6-5A	280.899	2	1	280	3
116	FX6-5A	280.899	2	1	260	3
117	FX6-5B	280.899	2	1	340	4
118	FX6-5B	280.899	2	1	320	2
119	FX12-6	582.153	6	1	300	2
120	FX12-6	582.153	6	2	300	0
121	FX12-6	582.153	6	3	320	4
122	FX12-6	582.153	6	4	320	3
123	FX12-6	582.153	6	5	300	3
124	FX12-6	582.153	6	6	300	2
125	FX12-1	150.304	5	1	290	3
126	FX12-1	150.304	5	2	300	3
127	FX12-1	150.304	5	3	290	3
128	FX12-1	150.304	5	4	280	2
129	FX12-1	150.304	5	5	260	3

130	FX9-5	119.6	0	1	300	NA
131	FX12-5	61.75	3	1	280	4
132	FX12-5	61.75	3	2	310	2
133	FX12-5	61.75	3	3	330	3
134	FX7-7	97.944	3	1	320	0
135	FX7-7	97.944	3	2	310	0
136	FX7-7	97.944	3	3	320	0
137	FX17-14	38.34	1	1	300	2
138	F26-5X	134.82	0	1	320	NA
139	FX19-4	220.48	1	1	290	2
140	FX7-4	190.736	4	1	340	2
141	FX7-4	190.736	4	1	300	2
142	FX7-4	190.736	4	1	300	3
143	FX7-4	190.736	4	1	240	3
144	FX1-2	364.1	1	1	320	2
145	FX9-17	268.92	5	1	260	2
146	FX9-17	268.92	5	2	280	2
147	FX9-17	268.92	5	3	300	3
148	FX9-17	268.92	5	4	260	2
149	FX9-18	403	3	1	320	1
150	FX9-18	403	3	2	300	2
151	FX9-18	403	3	3	280	1
152	FX9-14	145.86	2	1	320	3
153	FX12-18	303.282	2	1	300	3
154	FX12-18	303.282	2	2	280	2
155	FX12-13	348.936	4	1	350	3
156	FX12-13	348.936	4	2	310	2
157	FX12-13	348.936	4	3	300	2
158	FX12-13	348.936	4	4	310	2
159	FX12-14	383.04	3	1	300	3
160	FX12-14	383.04	3	2	310	2
161	FX12-14	383.04	3	3	340	3
162	FX12-14B	43.092	1	1	320	2
163	FX18-1	405.108	3	1	290	2
164	FX18-1	405.108	3	2	300	2
165	FX18-1	405.108	3	3	300	2
166	FX18-1B	32.89	1	1	310	4
167	FX9-13	295.659	2	1	330	2
168	FX9-13	295.659	2	2	350	3
169	FX4-9	934.912	5	1	320	3
170	FX4-9	934.912	5	1	320	3
171	FX17-15	244.218	2	1	300	1
172	FX17-15	244.218	2	2	300	2
173	FX4-9	934.912	5	3	280	2
174	FX4-9	934.912	5	4	310	2
175	FX4-9	934.912	5	5	340	2

176	FX13-5	212.8	2	1	340	0
177	FX13-5	212.8	2	2	300	1
178	FX13-9	347.776	5	1	310	1
179	FX13-9	347.776	5	2	300	1
180	FX13-9	347.776	5	3	290	1
181	FX13-9	347.776	5	4	320	1
182	FX13-9	347.776	5	5	300	1
183	FX9-1	209.952	2	1	300	3
184	FX9-1	209.952	2	2	330	2
185	FX12-3	190.35	2	1	330	3
186	FX12-3	190.35	2	2	320	2
187	FX14-3	258.552	4	1	260	1
188	FX14-3	258.552	4	2	280	0
189	FX14-3	258.552	4	3	280	0
190	FX14-3	258.552	4	4	280	0
191	FX17-13	256.23	2	1	290	1
192	FX17-13	256.23	2	2	320	1
193	FX14-1	867.3	8	1	280	1
194	FX14-1	867.3	8	2	300	1
195	FX14-1	867.3	8	3	290	0
196	FX14-1	867.3	8	4	320	1
197	FX14-1	867.3	8	5	280	1
198	FX14-1	867.3	8	6	280	1
199	FX14-1	867.3	8	7	280	1
200	FX14-1	867.3	8	8	300	2
201	FX14-1A	47.824	1	1	320	2
202	F8-3	24.15	2	2	310	3
203	FX12-7	203.456	1	1	320	3
204	FX23-1	102.114	1	1	290	3
205	FX7-1	190.736	5	1	310	3
206	FX7-1	190.736	5	2	340	2
207	FX7-1	190.736	5	3	300	3
208	FX7-1	190.736	5	4	320	2
209	FX7-1	190.736	5	5	310	3
210	FX9-17	268.92	5	5	250	2

oocyte	excluded_for_analysis
3387.42228	FALSE
NA	FALSE
883.572934	FALSE
NA	FALSE
NA	FALSE
NA	FALSE
113.097336	FALSE
977.428014	FALSE
174.947441	FALSE
0	FALSE
NA	FALSE
0	FALSE
0	FALSE
0	FALSE
896.139304	FALSE
0	FALSE
2064.02637	FALSE
0	FALSE
138.230077	FALSE
311.508547	FALSE
1610.26259	FALSE
682.511004	FALSE
1438.06404	FALSE
0	FALSE
235.619449	FALSE
592.190215	FALSE
125.663706	FALSE
428.827397	FALSE
NA	FALSE
NA	FALSE
176.714587	FALSE
481.449074	FALSE
0	FALSE
196.349541	FALSE
283.245994	FALSE
0	FALSE
0	FALSE

0	FALSE
0	FALSE
1032.01319	FALSE
157.079633	FALSE
176.714587	FALSE
235.619449	FALSE
0	FALSE
0	FALSE
100.530965	FALSE
0	FALSE
180.641578	FALSE
0	FALSE
0	FALSE
0	FALSE
NA	FALSE
49.4800843	FALSE
0	FALSE
75.3982237	FALSE
0	FALSE
232.477856	FALSE
157.079633	FALSE
137.444679	FALSE
237.582944	FALSE
176.714587	FALSE
196.349541	FALSE
1366.5928	FALSE
0	FALSE
75.3982237	FALSE
0	FALSE
282.743339	FALSE
0	FALSE
0	FALSE
272.140464	FALSE
0	FALSE
0	FALSE
0	FALSE
150.796447	FALSE
0	FALSE
0	FALSE
75.3982237	FALSE
0	FALSE
NA	FALSE
NA	FALSE
271.747765	FALSE
NA	FALSE
NA	FALSE

206.167018	FALSE
0	FALSE
NA	FALSE
NA	FALSE
NA	FALSE
NA	FALSE
NA	FALSE
NA	FALSE
143.138815	FALSE
580.703767	FALSE
438.252175	FALSE
452.389342	FALSE
0	FALSE
1911.16826	FALSE
0	FALSE
1113.49825	FALSE
137.444679	FALSE
2267.0518	FALSE
1242.30355	FALSE
0	FALSE
797.964534	FALSE
3092.89797	FALSE
1812.50261	FALSE
412.334036	FALSE
667.19574	FALSE
2213.25202	FALSE
726.493301	FALSE
0	FALSE
157.079633	FALSE
304.341788	FALSE
127.234503	FALSE
1299.44126	FALSE
1739.16606	FALSE
2068.34606	FALSE
268.409822	FALSE
750.840644	TRUE
0	TRUE
872.970059	TRUE
805.033118	TRUE
775.580686	TRUE
263.108385	TRUE
1368.949	FALSE
1656.79743	FALSE
1876.70891	FALSE
375.420322	FALSE
690.168636	FALSE

NA	FALSE
2507.77634	FALSE
1476.54855	FALSE
1370.1271	FALSE
0	FALSE
0	FALSE
0	FALSE
1311.22223	FALSE
NA	FALSE
1497.45977	FALSE
360.105058	FALSE
691.150384	FALSE
1452.5939	FALSE
552.134909	FALSE
1661.90251	FALSE
1696.46003	FALSE
2434.73431	FALSE
2087.98102	FALSE
2283.15246	FALSE
1256.63706	FALSE
1081.88597	FALSE
115.45353	FALSE
1201.65919	FALSE
2099.86017	FALSE
1055.37878	FALSE
3756.55942	FALSE
889.070721	FALSE
863.152582	FALSE
2223.4622	FALSE
1335.37323	FALSE
670.730032	FALSE
3560.20988	FALSE
962.603624	FALSE
2423.73873	FALSE
614.181364	FALSE
1224.73026	FALSE
1259.77865	FALSE
1527.10855	FALSE
2265.18648	FALSE
2572.17899	TRUE
1404.88097	TRUE
125.663706	FALSE
213.6283	FALSE
1121.15588	TRUE
757.12383	TRUE
1391.72555	TRUE

0	FALSE
119.380521	FALSE
395.840674	FALSE
424.115008	FALSE
564.112231	FALSE
367.566341	FALSE
196.349541	FALSE
1534.66801	FALSE
2072.27305	FALSE
2045.17682	FALSE
2020.24043	FALSE
147.262156	FALSE
0	FALSE
0	FALSE
0	FALSE
67.151543	FALSE
176.714587	FALSE
75.3982237	TRUE
196.349541	TRUE
0	TRUE
100.530965	TRUE
245.436926	TRUE
127.234503	TRUE
235.619449	TRUE
227.765467	TRUE
1062.25102	FALSE
530.14376	FALSE
2432.57446	FALSE
1521.70894	FALSE
2185.76309	FALSE
1426.57759	FALSE
1964.67351	FALSE
1024.9446	FALSE
1473.01426	FALSE
648.542533	FALSE

Dataset for nest level analysis of reproductive status versus nest volume

repro	Reproductive females in nest
nonrepro	Nonreproductive females in nest
vol	Nest volume (mm3)
females	Total number of females in nest

	id	repro	nonrepro	vol	females	excluded_for
1	F10-1	0	1	NA	1	NA
2	F10.0.1	0	1	47.4875	1	FALSE
3	F11-2	0	0	NA	0	NA
4	F11.2	2	0	NA	2	NA
5	F14.1	0	0	NA	0	NA
6	F16.0.2	3	1	281.799	4	FALSE
7	F17-8	0	0	NA	0	NA
8	F2.0.1	1	3	136.08	4	FALSE
9	F22.0.1A	0	0	204.867	0	FALSE
10	F26-3	1	0	NA	1	NA
11	F26-5X	0	0	134.82	0	FALSE
12	F28.1	2	0	NA	2	NA
13	F28.2	1	0	73.8675	1	FALSE
14	F33.1	1	0	NA	1	NA
15	F34.0.1	1	0	NA	1	NA
16	F36.1	0	2	NA	2	NA
17	F36.2	2	0	NA	2	NA
18	F36.3	0	0	64.8	0	FALSE
19	F37.1	0	1	NA	1	NA
20	F38.0.1	1	0	69.35	1	FALSE
21	F39.1	0	1	NA	1	NA
22	F40-1B	0	1	NA	1	NA
23	F47-3	0	0	NA	0	NA
24	F48.1	1	2	106.64325	3	FALSE
25	F48.2	1	1	75.992	2	FALSE
26	F6.0.1	1	0	129.36	1	FALSE
27	F7.0.1	0	0	69.356625	0	FALSE
28	F7.0.2	0	1	NA	1	NA
29	F8-1	1	0	NA	1	NA
30	F8-3	2	0	24.15	2	FALSE
31	F9.0.1	1	2	42.75	3	FALSE
32	FX.1	0	2	134.64	2	FALSE
33	FX1-1	2	0	380.16	2	FALSE
34	FX1-2	1	0	364.1	1	FALSE
35	FX11-2	4	0	248.009	4	FALSE
36	FX11-3	1	0	NA	1	NA
37	FX12-1	5	0	150.304	5	FALSE
38	FX12-10	0	0	NA	0	NA
39	FX12-12	0	0	NA	0	NA

40	FX12-13	4	0	348.936	4	FALSE
41	FX12-14	3	0	383.04	3	FALSE
42	FX12-14B	1	0	43.092	1	FALSE
43	FX12-16	0	0	NA	0	NA
44	FX12-18	2	0	303.282	2	FALSE
45	FX12-2	3	0	NA	3	NA
46	FX12-21	1	0	NA	1	NA
47	FX12-22	0	0	NA	0	NA
48	FX12-3	2	0	190.35	2	FALSE
49	FX12-5	3	0	61.75	3	FALSE
50	FX12-6	5	1	582.153	6	TRUE
51	FX12-7	1	0	203.456	1	FALSE
52	FX12-9	1	0	NA	1	NA
53	FX13-1	1	1	150.304	2	FALSE
54	FX13-12	2	0	NA	2	NA
55	FX13-4	2	0	NA	2	NA
56	FX13-4B	2	0	NA	2	NA
57	FX13-5	1	1	212.8	2	FALSE
58	FX13-9	5	0	347.776	5	FALSE
59	FX14-1	7	1	867.3	8	TRUE
60	FX14-1A	1	0	47.824	1	FALSE
61	FX14-3	1	3	258.552	4	FALSE
62	FX17-11	2	0	NA	2	NA
63	FX17-12	2	0	203.456	2	FALSE
64	FX17-13	2	0	256.23	2	FALSE
65	FX17-14	1	0	38.34	1	FALSE
66	FX17-15	2	0	244.218	2	FALSE
67	FX17-3	1	0	NA	1	NA
68	FX18-1	3	0	405.108	3	FALSE
69	FX18-1B	1	0	32.89	1	FALSE
70	FX18-2	1	0	NA	1	NA
71	FX18-3	2	0	NA	2	NA
72	FX18-5	1	0	NA	1	NA
73	FX19-4	1	0	220.48	1	FALSE
74	FX22-1	0	0	NA	0	NA
75	FX23-1	1	0	102.114	1	FALSE
76	FX24-1	1	0	NA	1	NA
77	FX25-1	4	0	NA	4	NA
78	FX26-2	3	0	NA	3	NA
79	FX26-4	2	0	NA	2	NA
80	FX27-5A	0	1	NA	1	NA
81	FX4-1	1	0	NA	1	NA
82	FX4-10	0	0	NA	0	NA
83	FX4-6	4	0	NA	4	NA
84	FX4-7	3	0	NA	3	NA
85	FX4-9	5	0	934.912	5	TRUE

86	FX5-1	1	1	NA	2	NA
87	FX5-3	0	0	NA	0	NA
88	FX5-4	0	1	NA	1	NA
89	FX6-1	1	0	NA	1	NA
90	FX6-5A	2	0	280.899	2	FALSE
91	FX6-5B	2	0	280.899	2	FALSE
92	FX6-7	1	1	240.352	2	FALSE
93	FX7-1	5	0	190.736	5	FALSE
94	FX7-1B	0	0	NA	0	NA
95	FX7-4	4	0	190.736	4	FALSE
96	FX7-7	0	3	97.944	3	FALSE
97	FX7-8	1	0	NA	1	NA
98	FX8-3	0	1	NA	1	NA
99	FX9-1	2	0	209.952	2	FALSE
100	FX9-10	1	1	139.36	2	FALSE
101	FX9-11	1	0	NA	1	NA
102	FX9-13	2	0	295.659	2	FALSE
103	FX9-14	2	0	145.86	2	FALSE
104	FX9-14B	1	0	NA	1	NA
105	FX9-15	3	1	277.35	4	FALSE
106	FX9-16	0	0	NA	0	NA
107	FX9-17	5	0	268.92	5	FALSE
108	FX9-18	3	0	403	3	FALSE
109	FX9-20	3	0	415.436	3	FALSE
110	FX9-3	3	0	NA	3	NA
111	FX9-5	0	0	119.6	0	FALSE
112	I1-12	1	0	55.176	1	FALSE
113	I1-13	0	1	NA	1	NA
114	I1-14	1	1	95.03	2	FALSE
115	I1-16	0	0	37.43	0	FALSE
116	I1-17	0	1	96.432	1	FALSE
117	I1-18	0	0	59.1465	0	FALSE
118	I1-2	1	0	83.232	1	FALSE
119	I1-20	0	0	12.42	0	FALSE
120	I1-21	1	0	53.7425	1	FALSE
121	I1-24	0	0	49.761	0	FALSE
122	I1-27	0	0	83.475	0	FALSE
123	I1-28	1	0	41.664	1	FALSE
124	I1-29	1	0	47.41125	1	FALSE
125	I1-3	1	0	56.43	1	FALSE
126	I1-33	1	0	114.71825	1	FALSE
127	I1-34	1	0	47.799	1	FALSE
128	I1-36	0	0	8.4245	0	FALSE
129	I1-37	2	5	109.8125	7	FALSE
130	I1-39	0	0	29.375	0	FALSE
131	I1-41	0	1	39.494	1	FALSE

132	I1-42	0	0	87.21	0	FALSE
133	I1-43	1	0	78.3475	1	FALSE
134	I1-45	0	1	NA	1	NA
135	I1-46	0	0	46.8	0	FALSE
136	I1-47	0	1	50.4	1	FALSE
137	I1-48	1	0	57.2565	1	FALSE
138	I1-49	0	1	78.32175	1	FALSE
139	I1-50	1	0	119.85	1	FALSE
140	I1-51	0	1	27.846	1	FALSE
141	I1-53	0	1	NA	1	NA
142	I1-54	0	0	44.655	0	FALSE
143	I1-56	0	2	24.31	2	FALSE
144	I1-57	1	0	45.6665	1	FALSE
145	I1-58	1	0	53.34	1	FALSE
146	I1-59	0	0	46.189	0	FALSE
147	I1-6	0	1	29.79675	1	FALSE
148	I1-60	0	0	NA	0	NA
149	I1-63	2	0	106.191	2	FALSE
150	I1-64	1	0	16.7025	1	FALSE
151	I1-68	1	0	89.28	1	FALSE
152	I1-69	0	0	49.8465	0	FALSE
153	I1-7	0	0	80.85	0	FALSE
154	I1-70	1	0	53.58	1	FALSE
155	I1-9	1	0	62.244	1	FALSE
156	I2-1	2	0	136.249	2	FALSE
157	I2-11	0	2	51.0245	2	FALSE
158	I2-14	2	0	158.055	2	FALSE
159	I2-2	1	0	98.3825	1	FALSE
160	I2-7	2	0	73.44	2	FALSE
161	I2-9	3	1	79.38	4	FALSE
162	I3-1	3	0	222.75	3	FALSE
163	I4-1	1	4	148.708	5	FALSE
164	I4-1A	0	1	NA	1	NA
